# Implementation of written asthma action plan among asthma patients in Türkiye: a multicenter, cross-sectional, descriptive study

**DOI:** 10.55730/1300-0144.5910

**Published:** 2024-08-19

**Authors:** Kurtuluş AKSU, Gürgün Tuğçe VURAL SOLAK, Pınar MUTLU, Levent Cem MUTLU, Görkem VAYIŞOĞLU ŞAHİN, Burcu YORMAZ, Aylin ÇAPRAZ, Ezgi ERDEM TÜRE, Yavuzalp SOLAK, Funda AKSU

**Affiliations:** 1 Division of Immunology and Allergy, Department of Chest Diseases, Ankara Atatürk Sanatorium Training and Research Hospital, University of Health Sciences, Ankara, Turkiye; 2 Department of Chest Diseases, Faculty of Medicine, Çanakkale 18 Mart University, Çanakkale, Turkiye; 3 Department of Chest Diseases, Faculty of Medicine, Tekirdağ Namık Kemal University, Tekirdağ, Turkiye; 4 Chest Diseases Clinic, Mardin Training and Research Hospital, Mardin, Turkiye; 5 Department of Chest Diseases, Faculty of Medicine, Selçuk University, Konya, Turkiye; 6 Department of Chest Diseases, Faculty of Medicine, Amasya University Sabuncuoğlu Şerafettin Training and Research Hospital, Amasya, Turkiye; 7 Chest Diseases Clinic, Ankara Etlik City Hospital, Ankara, Turkiye; 8 Şereflikoçhisar District Health Directorate, Ankara, Turkiye; 9 Department of Chest Diseases, Ankara Atatürk Sanatorium Training and Research Hospital, University of Health Sciences, Ankara, Turkiye

**Keywords:** Asthma, asthma action plan, asthma control level, asthma self-management plans, educational status

## Abstract

**Background/aim:**

There is currently no data from Türkiye on whether, following a diagnosis of asthma, patients are given an asthma action plan to implement. There is also no data on whether patients can manage their treatment based on the provided asthma action plans. The present study aimed to determine the use of asthma action plans in Türkiye and the awareness levels of patients about these plans.

**Materials and methods:**

A multicenter, cross-sectional descriptive study was conducted in the outpatient immunology, allergy, and pulmonology clinics of secondary and tertiary healthcare centers. Adult asthmatics filled out a case registration form regarding age, sex, educational status, duration of asthma, and smoking history. Subjects answered the Global Initiative for Asthma (GINA) assessment of control questions, indicated whether they had previously been offered an asthma action plan, and if they had received one, whether they benefited from it.

**Results:**

Data from 265 asthmatic adults (mean age: 48.4 years, standard deviation: 13.4 years), including 194 (73.2%) females were analyzed. The asthma of 212 (80.0%) patients was controlled, and was uncontrolled in 53 (20.0%) patients. An asthma action plan had been mentioned to 91 (34.3%) of the 265 patients. Among these 91 patients, 85 (93.4%) had been given a written asthma action plan, and 85 (93.4%) stated that they had benefited from the plan. The rate of being given an asthma action plan was significantly lower for illiterate patients than for patients with any education (p < 0.001).

**Conclusion:**

The rate of mentioning an asthma action plan to asthmatic patients is low. However, almost all patients who received an asthma action plan benefitted significantly from the plan. Thus, it is important to give asthmatic patients an action plan, ideally in written form.

## 1. Introduction

Asthma is a common, chronic respiratory disease affecting 1%–29% of the population in different countries.^1^ The asthma prevalence in Türkiye varies between 6.2% and 11.2% [1]. Asthma management should aim to achieve good control of patient symptoms, maintain normal activity levels, and minimize mortality, exacerbations, permanent airflow limitation, and other adverse effects of asthma.^1^ It is essential that patients with this chronic disease are given the education and skills needed to effectively manage their asthma.^1^ Asthma self-management plans are an important part of the long-term management of asthmatic patients. Asthmatic patients should be able to recognize changes in the severity of their asthma and make short-term changes in their treatment in response, according to predetermined guidelines. They also should know how and when to access medical care [2].

The Global Initiative for Asthma (GINA) recommends that a written asthma action plan is prepared for each patient based on changes in symptoms or peak expiratory flow (PEF). When and how to change reliever and control medications, use oral corticosteroids, and access medical care if symptoms do not respond to treatment should be included in the action plan.^1^

To date, no data has been collected in Türkiye on whether clinicians give an asthma action plan to patients following a diagnosis of asthma, or if one is given, whether patients can manage their treatment according to this plan. With this multicenter study covering many regions of the country, the current state of the implementation of asthma action plans in Türkiye is revealed.

## 2. Materials and methods

A multicenter, cross-sectional descriptive study was conducted in outpatient immunology, allergy, and pulmonology clinics of secondary and tertiary health-care centers. Asthmatics over the age of 18 who had been diagnosed with asthma for at least 1 year and used regular inhaler therapy were included in the study. Exclusion criteria were having a psychiatric illness, having a lung disease other than asthma (such as interstitial lung disease, cystic fibrosis, or bronchiectasis), and not giving consent to participate in the study.

Patients filled out a case registration form with demographic data including age, sex, educational status, years with asthma, and smoking history. To assess the implementation of asthma action plans, the patients answered questions on the GINA assessment of control and indicated whether they had ever been informed about an asthma action plan, whether they had ever been given an asthma action plan, whether they had complied with the plan, and whether they had benefited from the plan. Illiterate patients filled out the form with the help of their relatives.

Based on the approximate number of asthma patients reported by the Turkish Ministry of Health, the optimal sample size for this study was calculated using the Epi Info StatCalc program (CDC, Atlanta, GA, USA).^2^ With a prevalence of 50%, a margin of error of 5%, and a 99% confidence interval, the target sample size was determined to be 663 patients. The study was completed with a total of 265 participants.

### 2.1. Statistical analysis

SPSS 19 for Windows (IBM, Armonk, NY, USA) was used for the statistical evaluations. The statistical analysis included frequency tables, median (min–max) values and score averages from the GINA scale, a chi-square test, Fisher’s exact test, and the Kolmogorov–Smirnov test for compliance with normal distribution. Student’s t-test was used for dichotomous variables that fit the normal distribution, and the Mann–Whitney U test was used for those that did not fit the normal distribution. Statistical significance was delimited at p < 0.05.

## 3. Results

Within the scope of the study, 333 asthmatic patients filled in the case registration form. Among them, 68 patients who filled in the form incompletely were excluded. The remaining 265 patients were included for data analysis (Table 1). The mean age of the participants was 48.4 with a standard deviation of 13.4 years and 194 (73.2%) of them were female. The subjects had been followed up after a diagnosis of asthma for a median (min–max) of 4 (1–54) years. While 235 of the participants had at least a primary school education, 30 of them were illiterate. The asthma condition of 212 (80.0%) patients was under control, and that of 53 (20.0%) patients was not.

Ninety-one (34.3%) of the 265 patients included in the study had an asthma action plan explained to them, and 85 (93.4%) of those 91 stated that they benefited from the plan. The plan was given in a written form to 85 (93.4%) of the 91 patients to whom the asthma action plan was implemented. The GINA control scores of patients to whom an asthma action plan was explained were significantly lower than those to whom no asthma action plan was mentioned (p < 0.001). However, the rates of controlled and uncontrolled asthma were not significantly different between these two groups (p = 0.174). Illiterate patients had a significantly lower rate of receiving instructions about an asthma action plan than patients with any education (p < 0.001) (Table 2).

## 4. Discussion

Personal asthma action plans allow individuals with asthma to adjust their treatment according to changes in their disease, particularly those that indicate exacerbations. Additionally, patients’ involvement in treatment decisions within their plan improves their quality of life [3–6]. Ignacio-García JM et al. [3] evaluated the benefits of a 3-year asthma self-management education program with educational reinforcement in 63 adults with chronic asthma. They reported that, for adults with chronic asthma, an asthma self-management education program and educational supplement appeared to be effective in reducing asthma morbidity, improving lung function, and reducing oral steroid consumption [3]. Kaya et al. [4] evaluated the effects of a self-management plan in 63 adult asthmatic patients for one year. They showed that a personalized asthma plan can improve asthma control and reduce emergency visits, antibiotic treatments, systemic corticosteroid treatments, and unplanned doctor visits [4]. In a review evaluating the effectiveness of asthma self-management programs in asthmatics ages 16 and older, educational programs that allow patients to adjust their medications using a written action plan were shown to be more effective than other forms of asthma self-management [5]. Another study investigating the cost effectiveness of patient education in asthmatics showed that the education program significantly reduced the cost of the disease. Alongside the cost reduction, a 10-point improvement in the St George’s Respiratory Questionnaire score and a 5% improvement in forced expiratory volume in 1 s (FEV1) were observed [7].

International asthma guidelines, which are accepted all over the world, strongly recommend the implementation of written asthma action plans. Despite this recommendation, real-life data shows that there are implementation problems. A web-based study evaluating 1812 adult asthma patients in the United States found that 74% of patients with controlled asthma and 65% of patients with uncontrolled asthma never received an asthma action plan [8]. In Türkiye, there is no information about whether clinicians prepare and give asthma action plans to patients who are followed up with a diagnosis of asthma nor whether patients manage their treatments according to a plan.

With this multicenter study covering many regions of Türkiye, the aim was to investigate for the first time the applicability of the written asthma action plan in Türkiye. It was observed that approximately one-third of asthmatics were given an asthma action plan and approximately two-thirds were not. This finding is consistent with a web-based real-life study conducted in the United States and a study in primary healthcare settings conducted in Malaysian [8,9].

In this study, almost all of the patients who were told about the asthma action plan stated that they benefited from it. This information provided by the patients themselves makes an important contribution to the literature. In a recent similar study, it was found that providing a written asthma action plan led to a decrease in the frequency of acute asthma events in patients while improving treatment adherence, quality of life, and asthma control levels [10].

Another important finding is the significantly lower rate of asthma action plans given to illiterate patients than to patients with any education. Apter et al. found a significant association between health literacy and both quality of life and asthma control in patients with moderate and severe asthma. Their study concluded that interventions that consider and address patients’ literacy needs can lead to improved asthma control [11]. Together, these findings emphasize that extra attention should be paid to asthma education for illiterate asthmatics. For illiterate asthmatics, an illustrated asthma action plan can be a useful tool for self-medication [12].

This study has certain limitations, including not reaching the targeted sample size and the exclusion of primary health care centers. However, it is important to note that it features a patient population representative of the majority of the country and is the first to examine asthma action plan awareness in Türkiye.

The rate of mentioning an asthma action plan to patients with asthma is low. Almost all patients to whom an asthma action plan is recommended benefit significantly from the plan. Therefore, it is important to give patients with asthma an asthma action plan, and it is even more useful to give this plan in written form.

## Figures and Tables

**Table 1:** Demographic and clinical characteristics of participants (n = 265).

**Age, years, mean ± SD**	48.4 ± 13.4

**Sex, n (%)**	
Female	194 (73.2)
Male	71 (26.8)

**Asthma duration, years** *	8.0 (1–54)

**Smoking history, n (%)**	
Active smoker	64 (24.2)
Former smoker	58 (21.9)
Never smoker	143 (54.0)

**Cigarette packs per year** *	15.0 (1–70)

**Educational status, n (%)**	
Illiterate	30 (11.3)
Primary school and above	235 (88.7)

**GINA score** *	1 (0–4)

**GINA control level, n (%)**	
Controlled	212 (80.0)
Uncontrolled	53 (20.0)

* Values are given as median (min–max).

**Table 2:** Characteristic comparison of patients informed and not informed about an asthma action plan (n = 265).

	Informed patients (n = 91)	Uninformed patients (n = 174)	p

**Age, years, mean ± SD**	47.0 ± 12.0	49.2 ± 14.3	t = −1.268 0.206

**Sex, n (%)**			
Female	66 (34.0)	128 (66.0)	χ^2^ = 0.033
Male	25 (35.2)	46 (64.8)	0.857

**Educational status, n (%)**			
Illiterate	2 (6.7)	28 (93.3)	χ**^2^ = 11.490**
Primary school and above	89 (37.9)	146 (62.1)	**<0.001** **

**Asthma duration, years** *	10.0 (1–50)	7.0 (1–54)	**Z =** −**3.566 <0.001**

**GINA score** *	0.0 (0–4)	1.0 (0–4)	**Z =** −**4.783 <0.001**

**GINA control level, n (%)**			
Controlled	77 (36.3)	135 (63.7)	χ^2^ = 1.845
Uncontrolled	14 (26.4)	39 (73.6)	0.174

* Values are given as median (min–max);

t = Student’s t-test; χ^2^ = chi-square test; Z = Mann–Whitney U test;

** = Fisher’s exact test.

## Data Availability

The data set underlying this research is available upon reasonable request.

## Footnotes

1 Global Initiative for Asthma (2023). Global Strategy for Asthma Management and Prevention [online]. Website https://ginasthma.org/2023-gina-main-report [accessed 06 August 2024].

2 T.C. Sağlık Bakanlığı Türkiye Halk Sağlığı Kurumu (2013). Türkiye Kronik Hastalıklar ve Risk Faktörlerinin Sıklığı Çalışması Temel Bulgular [online] (in Turkish). Website https://hsgm.saglik.gov.tr/depo/birimler/kronik-hastaliklar-ve-yasli-sagligi-db/Dokumanlar/Raporlar/KRONIK_ARASTIRMA_TEMEL_BULGULAR.pdf [accessed 06 August 2024].
